# Mineralization induced by phosphorylated dry baker’s yeast

**DOI:** 10.1371/journal.pone.0239774

**Published:** 2020-09-25

**Authors:** Yoshihiro Ojima, Maya Kihara, Mami Yoshida, Koichi Igarashi, Tomoko Yoshida, Masayuki Azuma

**Affiliations:** 1 Department of Applied Chemistry and Bioengineering, Osaka City University, Osaka, Japan; 2 Research Center for Artificial Photosynthesis, Osaka City University, Osaka, Japan; Qatar University, QATAR

## Abstract

We found the mineralization of Cu during long-term Cu2+ adsorption onto dry baker’s yeast cells phosphorylated using sodium cyclo-triphosphate. Field emission scanning electron microscopy (FESEM) with energy-dispersive X-ray spectroscopy confirmed that the elemental composition of minerals were copper, phosphorus, and oxygen. Synchrotron-based X-ray absorption fine structure showed that the local structure around Cu atoms deposited on the mineral was almost identical to that of commercial copper (II) phosphate Cu_3_(PO_4_)_2_∙3H_2_O. However, the crystallinity was low, and the structure was slightly distorted. Time profile analysis using FESEM revealed that copper phosphate mineralization was first apparent on Day 3 of adsorption, whereas mineral formation plateaued at around Day 7. It seems that mineralization occurs by the local saturation of phosphate and Cu^2+^ on the yeast cells. Mineralization of the rare earth ion Dy^3+^ was also demonstrated during long-term adsorption. Mineralization on phosphorylated yeast cells appears to follow a common path for various types of metal ions and provides a promising technique for metal recovery via irreversible adsorption.

## Introduction

Commercial and domestic effluent can be contaminated by a wide range of pollutants, including heavy metals and rare earth elements. Such contaminants pose a significant hazard to the health of workers and the functioning of ecosystems. Hence, it is necessary to develop efficient and cost-effective approaches to remove such pollutants from effluent. Biosorption is a cost-effective and relatively simple technique for removing heavy metals or rare earth elements from effluent.

The term “biosorption” refers to the ability of certain types of microbially generated biomass to bind and concentrate metals. The biosorption process can be utilized to rapidly adsorb and concentrate metal ions from aqueous solutions, even those that are very dilute [1–3], and is therefore ideal for the treatment of contaminated effluent. The baker’s yeast *Saccharomyces cerevisiae* has been the subject of considerable research in biosorption. *S*. *cerevisiae* is ubiquitous worldwide, is generally regarded as safe, and has been shown to have the ability to remove toxic or precious metals, and radionuclides [4]. Yeast cells are a by-product of the fermentation industry and are therefore an accessible form of biomass for use as a bioadsorbent [4–6]. Many studies into the use of *S*. *cerevisiae* for biosorption have focused upon increasing its absorbance, to develop new applications for biomineralization [6–10]. Mineralization results in the irreversible removal of metal ions from solution. Compared with adsorbents that only cause reversible adsorption, mineralization has significant advantages in detoxifying harmful metal ions in the environment. To give an example, *S*. *cerevisiae* cells grown in high-phosphate medium have been shown to induce uranium mineralization from U(VI) in the solution [11]. Analysis of U(VI)-bearing minerals demonstrated the presence of H-autunite (HUO_2_PO_4_∙4H_2_O). The adsorbed U(VI) on the cell surface reacts with the P released from inside the yeast cells, resulting in the production of uranium phosphate by local saturation. Lead mineralization has also been reported using other types of yeast, such as *Kluyveromyces lactis* and *Pichia acacia*, grown in high-phosphate medium [12]. Phosphate mineralization using yeast cells has also been applied to rare earth elements, such as Ce, Yb, Sm, and Eu [13–15]. However, limited information is available and further research is needed for mineralization applications.

The use of non-living microbial biomass for biosorption offers some advantages over living microorganisms. Non-living microbes do not require nutrients and are not affected by toxic heavy metals. Moreover, non-living biomass can be stored for long periods [16, 17]. Physical and chemical biomass pretreatment methods can be used to improve the adsorption qualities of the biomass [8]. In our previous study, dry baker’s yeast that was phosphorylated using inorganic sodium cyclo-triphosphate (Na_3_P_3_O_9_) was found to adsorb heavy metal ions up to 1 mmol/g DCW due to the increased negative charge [18]. Phosphorylated yeast cells preferentially adsorbed the trivalent rare earth ions Nd^3+^ and Yb^3+^ from mixtures of heavy metals and rare earth ions. However, the phenomena underlying long-term adsorption have not been studied in detail, because metal ion adsorption by phosphorylated yeast cells is a rapid process, reaching completion within a few minutes.

In this study, the long-term adsorption of heavy metal and rare earth ions by dry phosphorylated baker’s yeast cells was investigated to evaluate a possibility of mineralization. The minerals formed on the surface of the phosphorylated yeast cells were characterized using field emission scanning electron microscopy (FESEM) with energy-dispersive X-ray spectroscopy (EDS), and X-ray absorption fine structure (XAFS). This is, to the best of our knowledge, the first report of the mineralizing of metal ions by non-living phosphorylated yeast cells.

## Materials & methods

### Phosphorylation of dry baker’s yeast cells

Commercial dry baker’s yeast (Nisshin Seifun Group, Tokyo, Japan) was phosphorylated following the methods reported in a previous study [18]. Briefly, yeast cells were washed five times with pure water and then fixed with 70% (v/v) ethanol for 2 h. The yeast cells were phosphorylated using a 20% sodium cyclo-triphosphate hexahydrate solution (Na_3_P_3_O_9_•6H_2_O (P_3m_)) at 50°C for 20 h. The pH of the solution was maintained at pH 12 by adding 3 M sodium hydroxide_(aq)_, with stirring. After the reaction had completed, the phosphorylated yeast cells were washed with distilled water and lyophilized.

### Mineralization of Cu^2+^ and Dy^3+^ by phosphorylated yeast cells

Mineralization was examined by suspending phosphorylated yeast cells at a concentration of 7.5 mg DCW/mL in 2 mL of Cu^2+^ or Dy^3+^ solution at a concentration of 1,000 ppm in a test tube. The test tube was shaken on a reciprocal shaker at 140 rpm at 30°C for 28 days. At each sampling time, the mixture was centrifuged at 3,000 g for 10 min and then at 17,800 g for 5 min to remove the cells and debris. The supernatant solutions collected after centrifugation were analyzed using a Varian Vista MPX simultaneous ICP optical emission spectrometer (Agilent Technologies, Santa Clara, CA, USA). The precipitated cells were used for subsequent analyses.

### FESEM observation with EDS analysis

The precipitated cells were washed and fixed in 2.5% glutaraldehyde for 2 h at 4°C and then dehydrated using a serial gradient of aqueous ethanol solutions. The samples were suspended in a 1:1 mixed solution of ethanol and t-butyl alcohol for 15 min and then immersed twice in t-butyl alcohol for 15 min each time. The samples were freeze-dried before being submitted to SEM observation.

The samples was observed using an FESEM, JSM 6500-FS (JEOL Ltd., Tokyo, Japan), and the composition of the samples was analyzed using an EDS attached to the FESEM instrument. Analyses were conducted using an accelerating voltage of 25 kV, with a dead time of 22%–30% and a collection time of 60 s. The mineral formation rate (%) was calculated from the FESEM images. The presence or absence of mineral attachment on each yeast cell was judged visually. Over 1,000 cells were analyzed for each sample.

### X-ray diffraction and XAFS analyses

The X-ray diffraction (XRD) data for the mineralized phosphorylated yeast cells were collected at 23.0°C with an X-ray diffractometer (Rigaku RAXIS-RAPID Imaging Plate, Cu-Kα, λ = 1.54178 Å). Cu K-edge and P K-edge XAFS measurements were performed at BL5S1 and BL6N1 of the Aichi Synchrotron Radiation Center, respectively. Cu K-edge XAFS spectra of all the samples were obtained by a transmission mode. P K-edge XAFS spectra of the sample and the chemical standard sample were obtained by a fluorescence and a total electron yield mode, respectively. For both analyses, copper (II) phosphate (Cu_3_(PO_4_)_2_∙3H_2_O) (FUJIFILM Wako Pure Chemical Corp., Osaka, Japan) was used as the chemical standard.

### Statistical analysis

Each result is presented as the mean ± the standard deviation for more than three independent experiments.

## Results and discussion

### Formation of needlelike material on phosphorylated yeast cells during long-term Cu^2+^ adsorption

In a previous study, we reported that the adsorption equilibrium of phosphorylated yeast cells was reached within 10 min [18]. In this study, the adsorption behavior of phosphorylated yeast cells was examined by extending the adsorption time. As a primary experiment, phosphorylated yeast cells were collected at 10 min or on Day 6 during Cu^2+^ adsorption and were observed using FESEM.

**Fig 1:**
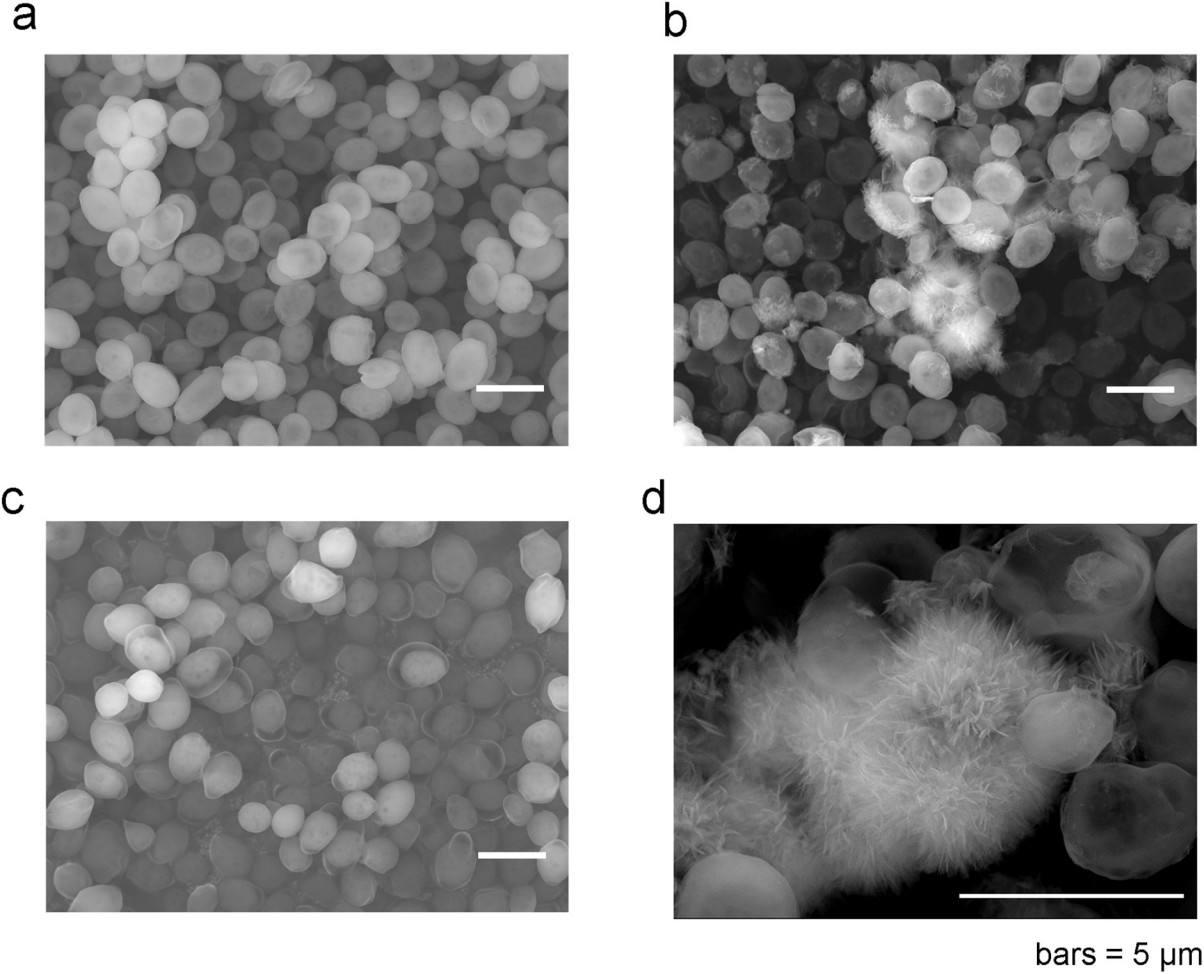
SEM images of dry baker’s yeast cells. (a) Phosphorylated yeast cells at 10 min during Cu^2+^ adsorption. (b) Phosphorylated yeast cells on Day 6 during Cu^2+^ adsorption. (c) Non-phosphorylated yeast cells on Day 6 during Cu^2+^ adsorption. (d) Zoomed image of phosphorylated yeast cells on Day 6 during Cu^2+^ adsorption.

Fig 1a shows the FESEM image of phosphorylated yeast cells collected at 10 min during Cu^2+^ adsorption. A clear elliptical from of each yeast cell was observed, consistent with previous observations that there were no significant changes in cell shape because of phosphorylation [18]. At Day 6 of adsorption, some material partially covered the yeast cells (Fig 1d). Zoomed images revealed that the yeast cells were covered by the needlelike material. In contrast, as shown in Fig 1c, no material was observed on the non-phosphorylated yeast cells, even after the cells had adsorbed Cu^2+^ for 6 days. These results suggest that the formation of needlelike material is a phenomenon unique to phosphorylated yeast during long-term Cu^2+^ adsorption. Therefore, the subsequent detailed analysis of mineralization focused on the phosophorylated yeast cells. Ohnuki et al. reported the formation of a very similar needlelike material on living *S*. *cerevisiae* cells grown in a high-phosphate medium containing uranium. This material was identified as uranyl phosphate mineralization [11]. Therefore, it seems likely that the needlelike material observed on the phosphorylated yeast cells was composed of a mineral.

### Elemental composition analysis of needlelike materials formed on phosphorylated yeast cells

**Fig 2:**
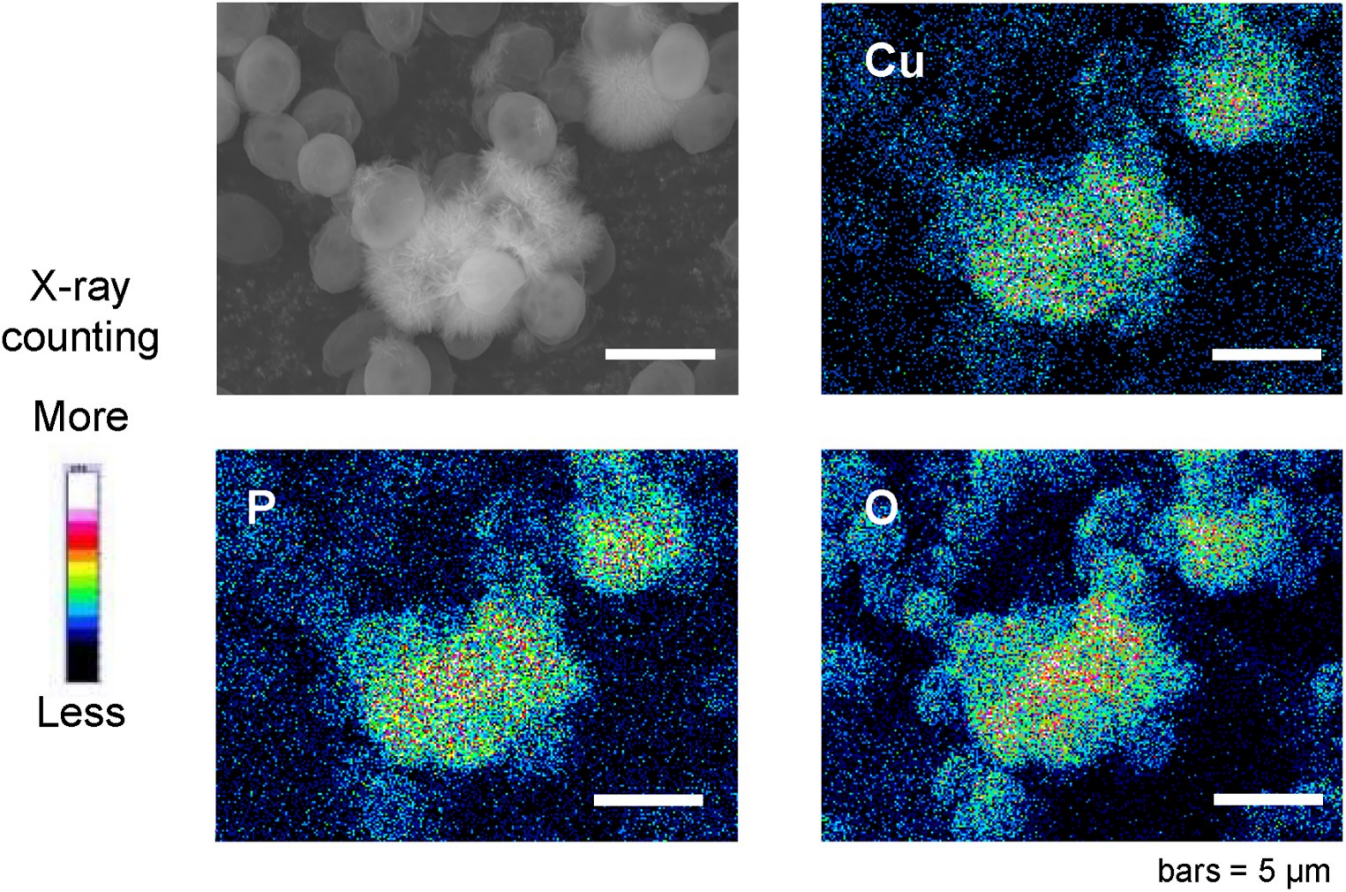
SEM image and two-dimensional localization of copper, phosphorus, and oxygen on phosphorylated yeast cells analyzed by FESEM-EDS. Yeast cell samples were collected on Day 6 of Cu^2+^ adsorption.

The elemental composition analysis of the needlelike material was analyzed using samples of Cu^2+^-adsorbed phosphorylated yeast cells collected on Day 6, freeze-dried, and placed in the vacuum chamber of a standard FESEM equipped with EDS. EDS spectra were recorded from different areas of the sample. The peaks of three elements—copper, phosphorus, and oxygen—were detected in EDS spectra from the region containing the needlelike material. Typical results of the two-dimensional localization of the three elements are shown in Fig 2. Copper was localized at the region of needlelike material. Areas containing high densities of phosphorus and oxygen overlapped and occupied the same region as the copper. Elemental composition analysis suggests that the needlelike material formed on the phosphorylated yeast cells was a copper phosphate mineral.

### Structural analysis of the mineral formed on the phosphorylated yeast cells

Elemental composition analysis showed that needlelike material was a copper phosphate mineral composed of copper, phosphorus, and oxygen. The crystal structure of the copper phosphate mineral was investigated using XRD and XAFS. Commercial copper (II) phosphate, which is mainly composed of Cu_3_(PO_4_)_2_∙3H_2_O, was used as the standard. As shown in S1 Fig, the XRD pattern of the sample did not show any diffraction peak, in contrast to the standard. This result suggests that the copper phosphate mineral formed on the phosphorylated yeast cells is an amorphous or low-crystalline solid.

**Fig 3:**
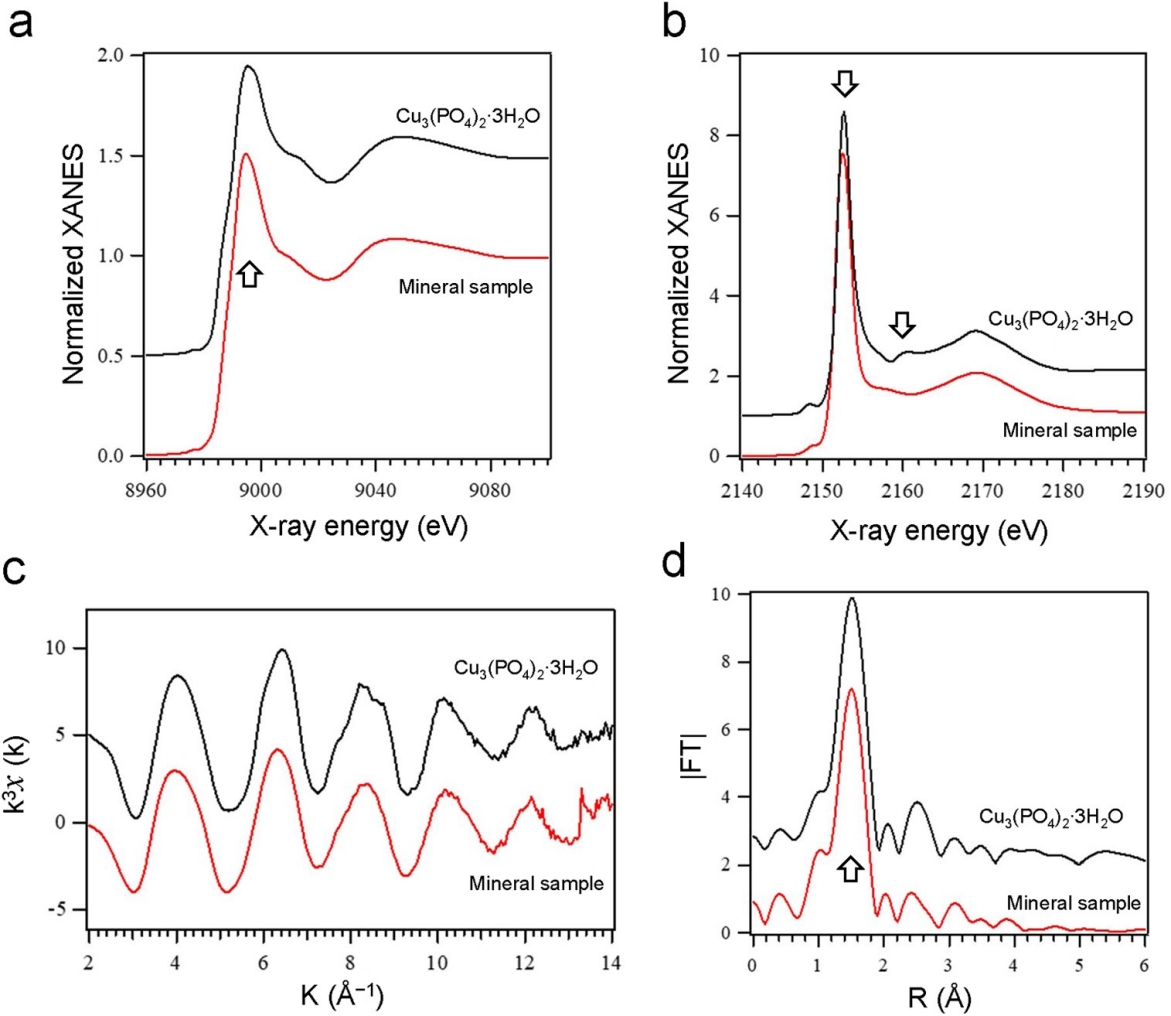
XANES and EXAFS spectra of the minerals formed on the phosphorylated yeast cells. Yeast cell samples were collected on Day 6 of Cu^2+^ adsorption. Copper (II) phosphate (Cu_3_(PO_4_)_2_∙3H_2_O) was used as the standard. (a) Cu K-edge XANES (b) P K-edge XANES (c) Cu K-edge EXAFS (d) Radial structure function (RSF).

The structure of the sample was further investigated by XAFS analysis. Since XAFS provides local structure of the sample, it was applied to the analysis of amorphous or low-crystalline solids. As shown in XANES spectra in Fig 3, the Cu K-edge and P K-edge appeared at the same energy positions for the sample and the standard, suggesting that the valences of Cu and P were the same for both samples. Cu K-edge EXAFS and RSF (radial structure function) obtained by Fourier transform of the EXAFS of the sample were very similar to those of the standard, indicating that the Cu species present in the sample was almost identical to Cu_3_(PO_4_)_2_∙3H_2_O. The amplitude of EXAFS oscillation and the peak intensity of around 1.5 Å in RSF were slightly higher for the standard. Moreover, in the P K-edge XANES, a small peak near 2,160 eV indicative of the high crystallinity was observed only in the standard. From these results, it can be concluded that the local structure around the Cu atoms deposited on the sample was almost the same as that of Cu_3_(PO_4_)_2_∙3H_2_O; however, the crystallinity was low, and the structure was slightly distorted.

### Time profile analysis and mechanism of mineralization

In the experiment described above, mineralization on the phosphorylated yeast cells was clearly observed on Day 6 of Cu^2+^ adsorption. To investigate the details of mineralization, a time profile analysis of mineralization was conducted over the period from Days 1 to 28. Mineralization was evaluated using FESEM observation (S2 Fig). At Days 1 and 2 of Cu^2+^ adsorption, no mineralization was visible using FESEM observation. Mineralization was first confirmed on Day 3, suggesting that it takes 3 days for the mineral deposit to grow to a visible size. Mineralization proceeded until Day 7, and large clusters of minerals involving many yeast cells subsequently appeared, as indicated by arrows (S2 Fig).

**Fig 4:**
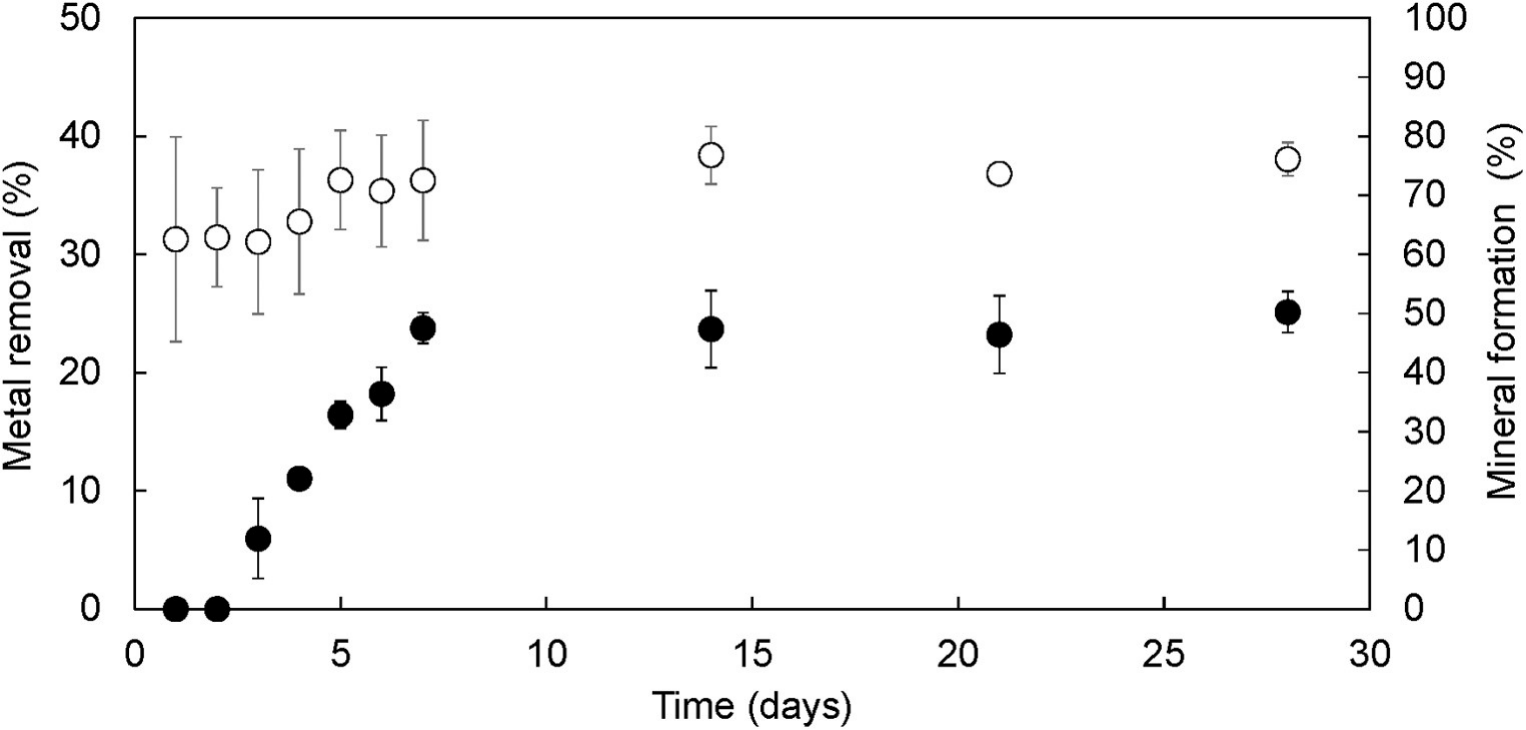
Time profiles of metal removal in the Cu^2+^ solution (%) (open circle) and mineral formation (%) (closed circle) during long-term adsorption of Cu^2+^ using phosphorylated yeast cells. Mineral formation was defined by visual assessment of the presence or absence of mineral attachment on each yeast cell.

The extent of mineral formation was calculated by visually determining the presence or absence of mineral on each yeast cell. Over 1,000 cells were analyzed from each sample. Fig 4 shows the time profiles of mineral formation and Cu^2+^ removal. Cu^2+^ removal (%) was determined by measuring the Cu^2+^ concentration in the solution. Mineral formation, as assessed by SEM observation, remained zero until Day 2. In a previous study, we reported that adsorption equilibrium of Cu^2+^ onto phosphorylated yeast cells was reached within 10 min [18]. These data indicate that it may take 3 days for a mineral deposit to grow to a size that can be confirmed by observation. Other researchers have also reported that uranium or lead phosphate mineralization took 48 h in living yeast cells grown in high-phosphate medium [11, 12], as estimated using SEM. Therefore, it seems that copper phosphate mineralization in yeast cells takes about 3 days to become visible. When mineralization was first observed on Day 3, mineral formation was approximately 5% and linearly increased afterward. Mineral formation reached 47.5% by Day 7 and did not increase subsequently. At Day 28, mineral formation was still 50.2%. These results indicated that mineralization was completed in approximately 1 week.

Cu^2+^ removal on Day 1 was 31.2 ± 8.7%, and the calculated adsorption capacity was 0.66 ± 0.22 mmol/g DCW. Although the standard deviation is large, this value is a little lower than that reported in our previous paper (1.08 mmol/g DCW) [18]. One of the reasons for this discrepancy is the lower molar ratio of Cu^2+^ against dry cell weight. This parameter was optimized for the mineralization experiment. Cu^2+^ removal did not significantly increase over time, reaching 36.3 ± 5.1% on Day 7. By Day 28, Cu^2+^ removal was 38.1 ± 1.4%, and calculated adsorption capacity was 0.80 ± 0.04 mmol/g DCW. Since mineral formation increased linearly from Days 3 to 7, it appears that mineral was formed by changing the state of metal ions already adsorbed on the yeast cells rather than by growing because of the continuous adsorption of new metal ions. A similar phenomenon was observed in the case of uranyl phosphate (HUO_2_PO_4_∙4H_2_O) mineralization [11]. In that report, it was concluded that adsorbed U(VI) on the yeast cell surface reacted with the P released from inside the cells, resulting in the production of uranium phosphate by local saturation. In phosphorylated yeast cells, metal ions are adsorbed on the surface of yeast cells via modified phosphate groups. Furthermore, the released P from the phosphorylated cells was detected in the solution during mineralization. Therefore, similar local saturation may occur between released phosphorus and adsorbed copper ions on the yeast cell surface, followed by mineral formation.

### Mineralization of rare earth ions on phosphorylated yeast cells

In the previous sections, the process of copper phosphate mineralization on phosphorylated yeast cells was described. In a previous study, it was reported that phosphorylated yeast cells selectively adsorbed rare earth ions from a solution containing heavy metals and rare earth ions, because trivalent positively charged ions were adsorbed preferentially over divalent ions [18]. Only a few reports have described the mineralization of rare earth ions using yeast cells [13–15]. In those reports, yeast cells grown in high-phosphate medium were also used for mineralization of rare earth ions, such as Ce^3+^, Yb^3+^, Sm^3+^, and Eu^3+^, as well as heavy metal ions such as Cu^2+^ and Pb^2+^. We investigated the ability of phosphorylated yeast cells to induce mineralization of dysprosium during adsorption of Dy^3+^, using the same procedure as was used for Cu^2+^.

**Fig 5:**
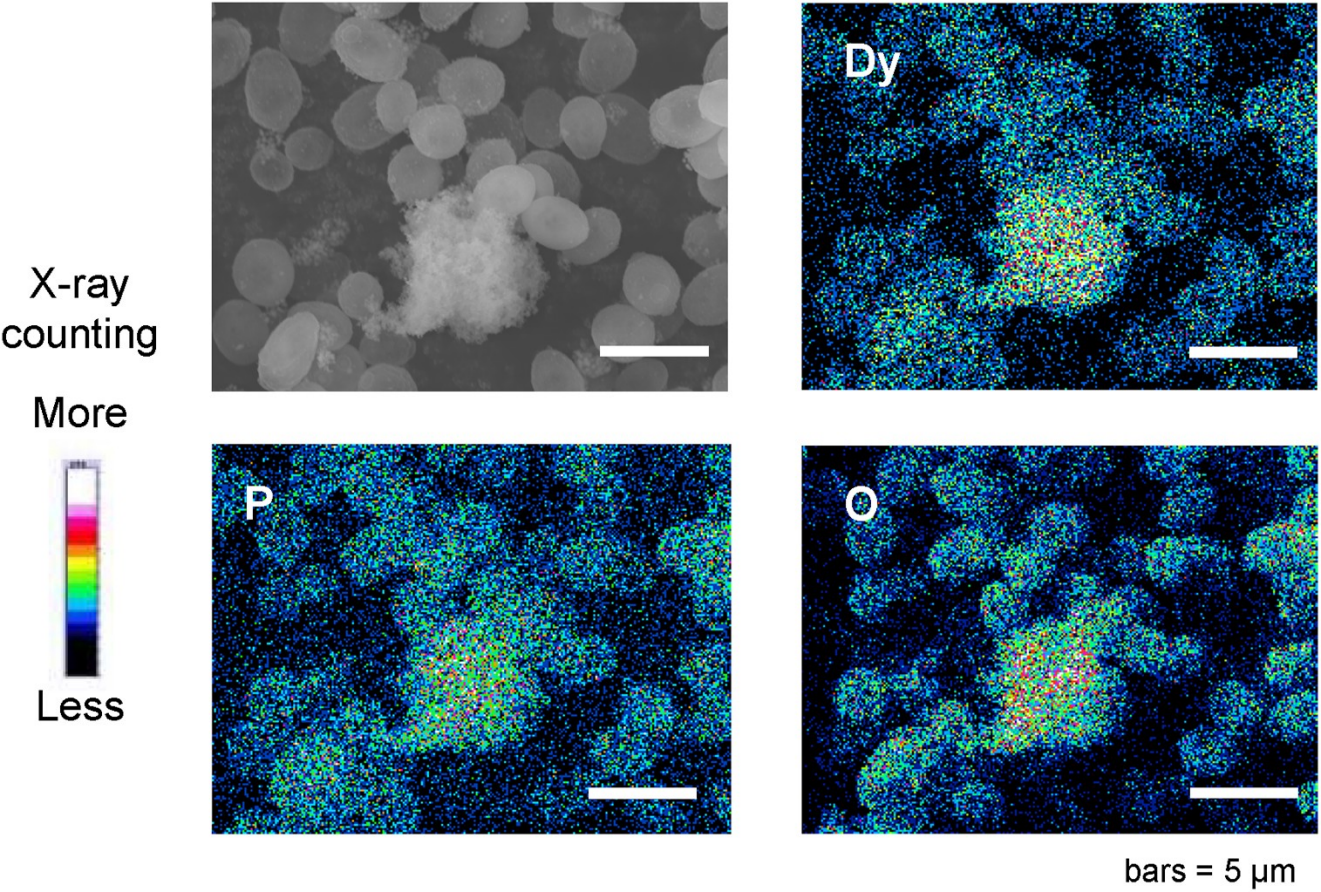
SEM image and two-dimensional localization of dysprosium, phosphorus, and oxygen on phosphorylated yeast cells, analyzed using FESEM-EDS. Yeast cell samples were collected on Day 6 of Dy^3+^ adsorption.

Dy^3+^-adsorbed phosphorylated yeast cells collected on Day 6 were freeze-dried and placed in the vacuum chamber of a standard FESEM equipped with an EDS. FESEM images showing typical results of the two-dimensional localization of dysprosium, phosphorus, and oxygen are shown in Fig 5. As with copper, mineral formation was observed during long-term adsorption of Dy^3+^ on phosphorylated yeast cells. The localization of the dysprosium element was confirmed to be the region of mineral formation, indicating that one of the components of the material is dysprosium. Areas containing high densities of phosphorus and oxygen overlapped with the same region, as seen in the case of copper phosphate minerals. These results suggest that mineralization on phosphorylated yeast cells has a common modality for different types of metal ions, including heavy metals and rare earth ions. Mineralization by phosphorylated yeast cells is a promising technique for the irreversible adsorption of a range of different metal ions.

## Conclusions

Mineralization of metal ions during long-term adsorption on dry baker’s yeast cells phosphorylated using sodium cyclo-triphosphate (Na_3_P_3_O_9_) was confirmed in these experiments. FESEM-EDS analysis confirmed that copper phosphate was the mineral adsorbed. XAFS analysis showed that the local structure around Cu atoms deposited on the mineral is almost the same as copper (II) phosphate. However, the crystallinity of the deposited minerals was low, and the structure slightly distorted. Time profile analysis using FESEM observation revealed that copper mineralization was first visible on Day 3 of adsorption, and the mineral formation plateaued at around Day 7. Mineralization occurred by the local saturation of phosphate and Cu^2+^ that primarily absorbed on the phosphorylated yeast cells. Finally, mineralization of the rare earth ion Dy^3+^ was also confirmed. Mineralization by phosphorylated yeast cells is a promising technique for irreversible adsorption of various metal ions. Further development of this technology could therefore lead to the production of efficient, cost-effective approaches to the remediation of contaminated effluent in a range of applications.

## Supporting information

S1 Fig XRD spectra of the minerals formed on the phosphorylated yeast cells. Yeast cell samples were collected on Day 6 of Cu^2+^ adsorption. Copper (II) phosphate (Cu_3_(PO_4_)_2_∙3H_2_O) was used as the standard.
(PDF)

S2 Fig SEM images of phosphorylated yeast cells at each sampling time during long-term adsorption of Cu^2+^. (PDF)

## References

[1] Li PS, Tao HC. Cell surface engineering of microorganisms towards adsorption of heavy metals. Crit Rev Microbiol. 2015; 41(2):140–149. 10.3109/1040841X.2013.813898 23915280

[2] Das N. Recovery of precious metals through biosorption—A review. Hydrometallurgy. 2010; 103(1–4):180–189. 10.1016/j.hydromet.2010.03.016

[3] Das N, Das D. Recovery of rare earth metals through biosorption: An overview. J Rare Earth. 2013; 31(10):933–943. 10.1016/S1002-0721(13)60009-5

[4] Wang J, Chen C. Biosorption of heavy metals by *Saccharomyces cerevisiae*: A review. Biotechnol Adv. 2006; 24(5):427–451. 10.1016/j.biotechadv.2006.03.001 16737792

[5] Park JK, Lee JW, Jung JY. Cadmium uptake capacity of two strains of *Saccharomyces cerevisiae* cells. Enzyme Microb Technol. 2003; 33(4):371–378. 10.1016/S0141-0229(03)00133-9

[6] Xin S, Zeng Z, Zhou X, Luo W, Shi X, et al Recyclable *Saccharomyces cerevisiae* loaded nanofibrous mats with sandwich structure constructing via bio-electrospraying for heavy metal removal. J Hazard Mater. 2017; 324(Pt B):365–372. 10.1016/j.jhazmat.2016.10.070 27847250

[7] Geva P, Kahta R, Nakonechny F, Aronov S, Nisnevitch M. Increased copper bioremediation ability of new transgenic and adapted *Saccharomyces cerevisiae* strains. Environ Sci Pollut Res Int. 2016; 23(19):19613–19625. 10.1007/s11356-016-7157-4 27392627

[8] De Rossi A, Rigon MR, Zaparoli M, Braido RD, Colla LM, et al Chromium (VI) biosorption by *Saccharomyces cerevisiae* subjected to chemical and thermal treatments. Environ Sci Pollut Res Int. 2018; 25(19):19179–19186. 10.1007/s11356-018-2377-4 29808404

[9] Qiu L, Feng J, Dai Y, Chang S. Biosorption of the strontium ion by irradiated *Saccharomyces cerevisiae* under culture conditions. J Environ Radioact. 2017; 172:52–62. 10.1016/j.jenvrad.2017.03.007 28324686

[10] Shen Y, Zheng X, Wang X, Wang T. The biomineralization process of uranium(VI) by *Saccharomyces cerevisiae*—transformation from amorphous U(VI) to crystalline chernikovite. Appl Microbiol Biotechnol. 2018; 102(9):4217–4229. 10.1007/s00253-018-8918-4 29564524

[11] Ohnuki T, Ozaki T, Yoshida T, Sakamoto F, Kozai N, et al Mechanisms of uranium mineralization by the yeast Saccharomyces cerevisiae. Geochimica et Cosmochimica Acta. 2005; 69(22):5307–5316. 10.1016/j.gca.2005.06.023

[12] Liang X, Laszlo C, Geoffrey MG. Lead bioprecipitation by yeasts utilizing organic phosphorus substrates. Geomicrobiology journal. 2016; 33(3–4):294–307. 10.1080/01490451.2015.1051639

[13] Jiang M, Ohnuki T, Utsunomiya S. Biomineralization of Middle Rare Earth Element Samarium in Yeast and Bacteria Systems. Geomicrobiology Journal. 2018; 35(5):375–384. 10.1080/01490451.2017.1377320

[14] Jiang M, Ohnuki T, Kozai N, Tanaka K, Suzuki Y, et al Biological nano-mineralization of Ce phosphate by Saccharomyces cerevisiae. Chemical Geology. 2010; 277(1):61–69. 10.1016/j.chemgeo.2010.07.010

[15] Jiang M, Ohnuki T, Tanaka K, Kozai N, Kamiishi E, et al Post-adsorption process of Yb phosphate nano-particle formation by Saccharomyces cerevisiae. Geochimica et Cosmochimica Acta. 2012; 93:30–46. 10.1016/j.gca.2012.06.016

[16] Vijayaraghavan K, Jegan J, Palanivelu K, Velan M. Biosorption of cobalt(II) and nickel(II) by seaweeds: batch and column studies. Sep Puri Technol. 2005; 44(1):53–59. 10.1016/j.seppur.2004.12.003

[17] Amini M, Younesi H, Bahramifar N. Biosorption of U(VI) from aqueous solution by *Chlorella vulgaris*: equilibrium, kinetic, and thermodynamic studies. J Environ Eng. 2013; 139(3):410–421. 10.1061/(ASCE)EE.1943-7870.0000651

[18] Ojima Y, Kosako S, Kihara M, Miyoshi N, Igarashi K, et al Recovering metals from aqueous solutions by biosorption onto phosphorylated dry baker’s yeast. Scientific Reports. 2019; 9(1):225 10.1038/s41598-018-36306-2 30659210 PMC6338781

