# Supplementary figures and images for: Mineralization induced by phosphorylated dry baker’s yeast
Source: PLoS One. 2020 Sep 25;15(9):e0239774. doi: 10.1371/journal.pone.0239774 (PMC7518573; doi:10.1371/journal.pone.0239774)

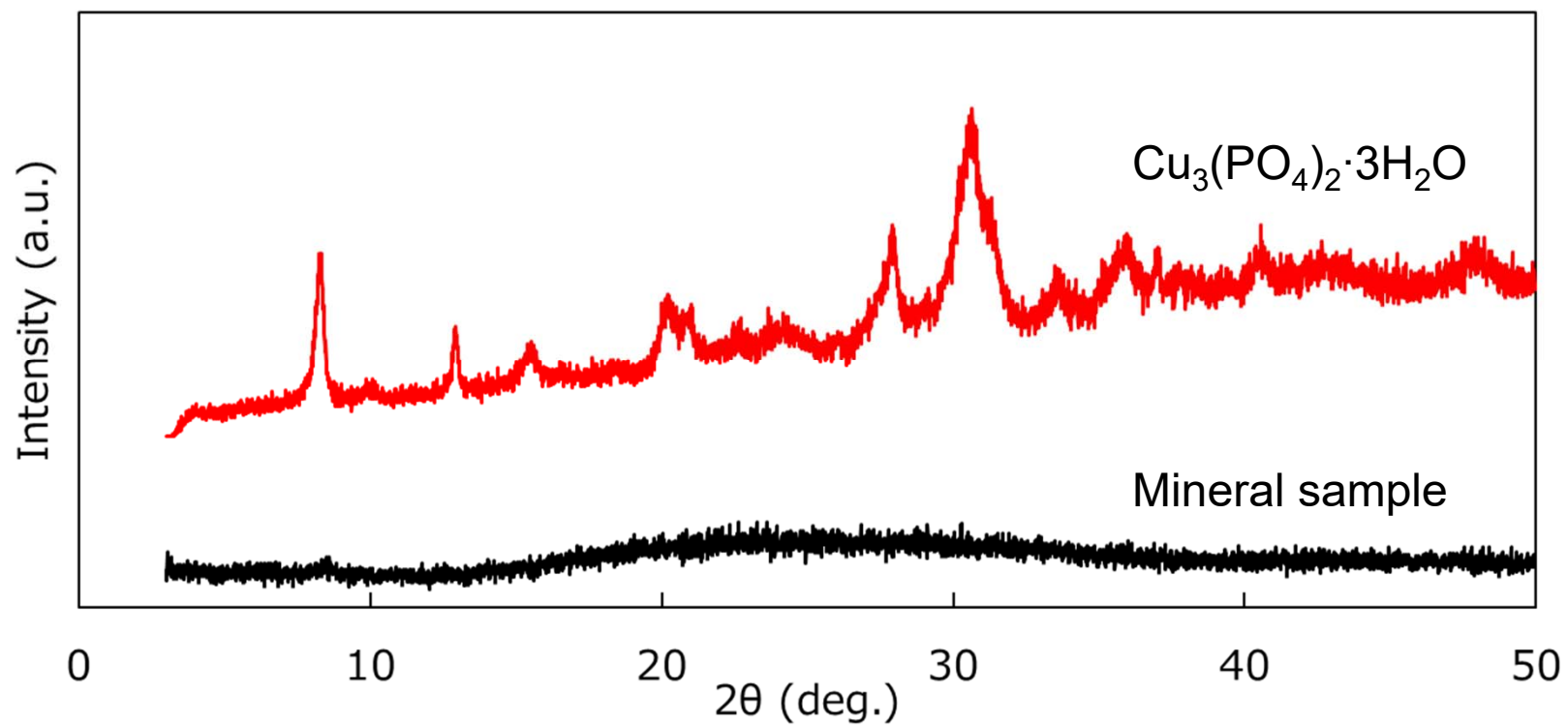

Supplement: S1 Fig — Yeast cell samples were collected on Day 6 of Cu2+ adsorption. Copper (II) phosphate (Cu3(PO4)2∙3H2O) was used as the standard. (PDF) [file pone.0239774.s001.pdf]

**day 1**

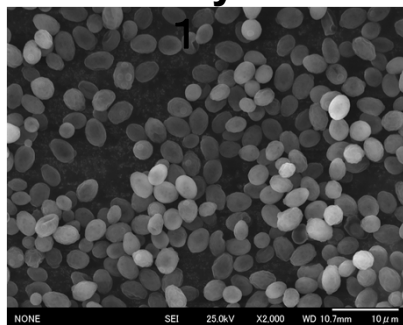

**day 2**

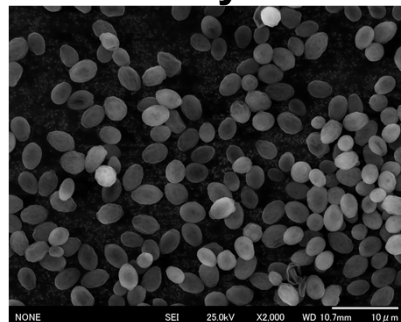

**day 3**

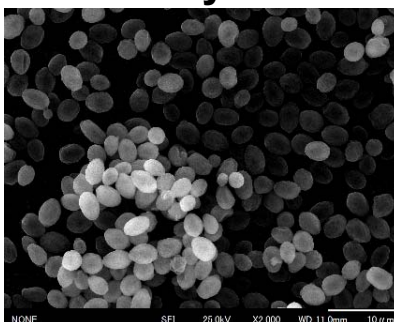

**day 4**

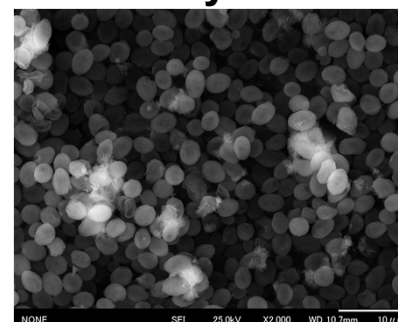

**day 5**

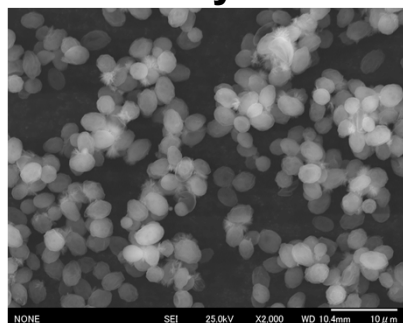

**day 6**

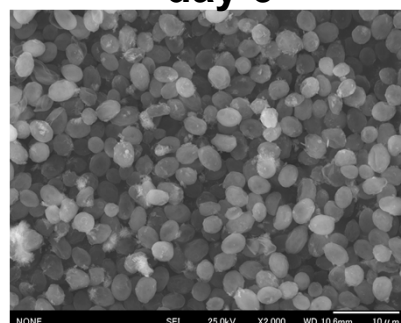

**day 7**

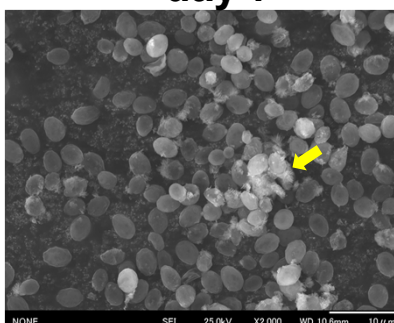

**day 14**

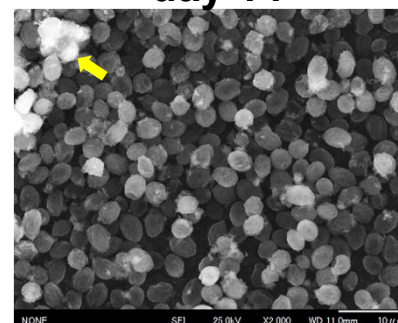

**day 21**

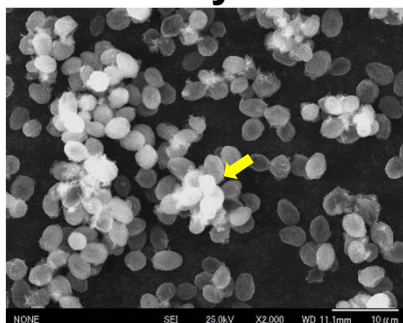

**day 28**

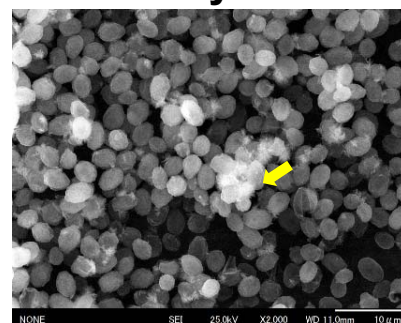

—  
10 μm

Supplement: S2 Fig — (PDF) [file pone.0239774.s002.pdf]
